# Collectanea

**Published:** 1832-10

**Authors:** 


### COLLECTANEA.

**Floriferis ut apes in saltibus omnia libant,**
**Omnia nos, itidem, depascimur aurea dicta.**

### PRACTICAL MEDICINE.

*Cherry-laurel Water in Cholera.*—Dr. Dudon, of Batignolles, states that he has employed the cherry-laurel water externally, with much benefit, to relieve the epigastric pains which so frequently follow the vomitings in cholera. He makes an almond poultice, to which the cherry-laurel water is added, and, applying this to the seat of the pain, in a very few minutes the gastralgia almost always is completely relieved. He likewise proposes to administer the same medicine internally.—*Gazette Medicale.)*

*Cases of* Choleraphobia; *with Remarks on the Effects of Fear as producing Disease during the present Epidemic Cholera.* By Dr. Tellier.

Case I. A woman having died in a few hours with a violent attack of cholera, in a district where the disease had been previously unknown, her neighbour, a nervous female, was greatly alarmed, and suddenly seized with shiverings, universal tremblings, and inexpressible uneasiness. She was put to bed, made warm, and had warm drinks administered; and all untoward symptoms disappeared in a few hours. Some days afterwards she was taken with a serious looseness in the bowels, about which, however, she did not feel alarmed; and in the night she was suddenly seized with violent cholera, and died.

Case II. A man of a tolerably strong constitution, who had been bled a few days before for palpitations of the heart, fancied, all of a sudden, that he was seized with cholera, because he had some shiverings and cramps, and especially because cholera was in the country. He went to bed between the blankets, had bottles of hot water, and drank abundantly of very warm tea. His countenance was excited, skin hot and moist, pulse frequent and hard; he had neither vomiting nor purging, urine secreted as usual, tongue natural, and intellects more active than usual. Some hours afterwards, he became more and more light-headed; he jumped out of bed stark naked, exclaiming that he had not an hour to live. His physician, arriving at the moment, spoke to him in an authoritative tone, which dissipated his alarm: he went to bed again, slept quietly, and on the following day was perfectly well.

Case III. A chambermaid, twenty-four years old, who had been ailing for several days, was seized, a few minutes after she had taken some coffee at breakfast, with much uneasiness and shivering: she fancied that she was attacked with cholera. She went to bed, made herself as warm as possible, and drank hot chamomile tea. Pulse small, and 108 per minute; heart beating tumultuously; skin hot, tongue natural, respiration somewhat embarrassed. There was neither vomiting nor purging, but her spirits were greatly depressed. Some ordinary medicines, with an opiate, were administered. She slept soundly, and on the following day was so well that she remained up a long while, and subsequently merely suffered with common diarrhœa.

Case IV. A woman, thirty-five years old, lately cured of an ordinary bowel complaint, after being exposed to cold in the evening, and washing her hands in cold water, and eating a hard egg, felt cold, and went to bed, but was shortly obliged to get up to relieve her bowels: she then was seized with shivering, and felt convinced that she was attacked with cholera. Her pulse very quick, beating more than one hundred per minute; respiration short and difficult; she felt stifled, her heart beat violently, face red and hot, and tongue natural. She had neither vomiting nor purging. She was bled freely, and put on low diet, and demulcent medicines, with a slight opiate, prescribed; but the dyspnœa increased, and the pulse became still more accelerated. While the patient was loaded, or rather overloaded, according to her own desire, not only with bedclothes, but with all manner of garments, and hot bottles and warming pans continually applied, if the least breath of air flew on her, she complained of cold and shivered violently, even when her skin felt burning hot. During the day, leeches were applied to the pit of the stomach, but they were removed before they began to draw blood; and the mustard poultices ordered were not kept on. A grain and a half of opium, with three grains of digitalis, given in three divided doses, at intervals of three hours, had no effect on the pulse, and did not procure sleep. During the night the dyspnœa was greatly increased; the pulse rose to 140 per minute, and the heart beat with violence. She was again bled, and infusion of limeflowers, with orange-flower water, prescribed; but without relief. The intellects became disturbed, sensibility blunted, respiration still more difficult, and at length intermittent; and the patient died within thirty hours from the commencement of the attack.

From these cases, and many others he has observed, and an account of which he proposes to publish hereafter, Dr. Tellier concludes that even the most violent fear cannot produce the "blue cholera," but bring on a particular disease, to which the name *phobia* (choleraphobia) may be given. The only known cause of this disease is fear; the symptoms are agitation, general uneasiness, disposition to shivering, skin hot, and often moist, pulse hard and frequent, countenance excited, tongue natural, absence of vomiting and purging, bowels sometimes confined, excitement of the nervous system and often cramps.

The diagnosis is easy, especially when the cause is known. The prognosis must be often unfavorable, since the disease so frequently has proved fatal.

The treatment is difficult to decide on. To calm the imagination is a thing in general impossible; the patient is pursuaded of the truth of his delusions, and the nervous system being in such a disordered state, he is no longer master of himself.—*Ibid.*

---

### MIDWIFERY.

### *Retroversion of the Uterus during Pregnancy: case communicated by* M. Rolland, *under the title of Hernia in the Bladder and Womb.*

A woman, twenty-five years old, and the mother of two children, being again pregnant, and advanced (according to her own calculation,) about four months, had experienced for some time a difficulty in passing her water, especially if she did not yield to the first desire for evacuation. On waking on the 6th of September, she complained of violent colicky pains, of a distressing weight about the hypogastrium, with an urgent want, without being able to pass her urine. This retention continued until the 13th; a little water only dribbling away from the overcharged bladder. Her bowels also had not acted. Fever supervened, the belly increased in size, and a roundish tumor, about the size of a child's head, made its appearance at the vulva. The labia majora became swelled; the perineum forced outwards; and the anus, half open, allowed a prolapsus of the rectum. The presence of strong abdominal pains made the patient believe she was in labour; her accoucheur likewise was deceived: but (as he said) not being able to make out any presentation, he did not remain with her during the whole continuance of the pains.

It was not until the 29th instant, seventeen days after the commencement of her illness,) that M. Rolland was consulted. On examining the tumor he felt a fluctuation. Having attempted to pass his finger into the vagina, he could penetrate no further than the arch of the pubis, and he could discover no trace either of the meatus urinarius, urethra, or os tincæ. On introducing two fingers into the rectum, he found the intestines pressed by the tumor against the hollow of the sacrum. The examination led him to suppose the case to be one of hernia of the bladder, which had thus fallen on the posterior part of the vagina.

Having placed the woman on her back, M. Roland endeavoured to reduce the hernia. The first efforts produced a copious discharge of urine, but the tumor did not yield in proportion to the efforts that were made. The patient was subsequently placed upon her knees and elbows, and then, introducing two fingers of the left hand into the rectum, raising at the same time, with the right hand, the tumor projecting from the vulva, he again attempted its reduction. The urine then gushed out abundantly in a stream the size of the little finger, and was ejected to the distance of six feet. This evacuation lasted for seven or eight minutes; after which M. Rolland succeeded in replacing the womb in its natural situation. The patient experienced immediately a feeling of relief; and the introduction of a catheter into the bladder brought away about five half-pints more of bloody urine. Confinement to bed, with the use of astringent injections, effected a rapid recovery. By the 25th the woman voided and retained her urine voluntarily. Pregnancy proceeded in the usual course, and she was safely delivered at the regular period.

On this case some remarks were made. The diagnosis had been erroneous twice. First, the tumor was mistaken for the head of a fœtus; but this could only have happened to a very ignorant practitioner. The second error appears to have been occasioned by the fluctuation perceptible in the tumor: this fluctuation was attributed to the presence of urine, and yet it was evidently produced by the liquor amnii. The relative positions of the bladder, uterus, and rectum, should be sufficient to prevent this last mistake, which could not be entertained without danger. A correct diagnosis must, in such a case, be of importance in regulating the plan of reduction. It is evident that the bladder should be emptied before any attempts are made towards replacing the uterus in its natural position; and in thus doing much difficulty would be avoided, and much pain spared to the patient. It is well known that forcible pressure made on the bladder, when so prodigiously distended, and for several days, might cause rupture; and such an accident, necessarily fatal, need never be feared after the evacuation of the urine.

M. Baudeloc considers that, with a little further enlargement of the uterus, its reduction would have been impossible without a previous discharge of the urine. The quantity evacuated was not measured, but it must have been very great.—(*Ac. de Med. Bull. des Sc. Med.*)

### MISCELLANEOUS.

### *On the Formation of Sulphur at the Solfatara, near Naples.*
(From the MSS. of Mr. A. S. Taylor.)

The Solfatara is an extinct volcanic crater in the immediate neighbourhood of Pozzuoli, and about two leagues distant from Naples. It is here that vast quantities of sulphur and alum are annually procured; and the works established for the refining and preparing of these substances are well worthy of inspection.

The Solfatara itself is a species of amphitheatre of an elliptical form, bounded by a ridge of volcanic tufa. The greater diameter of this elliptical crater measures, in a direction from NE. to SW., about 2,337 feet. The outer boundary of the ridge is about 6,805 feet in circumference, and 291 feet above the level of the sea. In various parts of this enclosed space, which presents a most remarkable appearance to the eye of the stranger, are to be seen, what are denominated in the language of the country, the "*fumarole*." They are nothing more than fissures or apertures in the soil, through which is seen to issue more or less abundantly, and with greater or less violence, the sulphuretted hydrogen gas. On the eastern border of the cratur the gas rises out of large apertures in the soil, with such force that small masses of lava are projected to a certain height in the air; especially if the larger blocks which intercept its passage be suddenly removed. The formation of the sulphur from the gas is an interesting process, and one to which I paid particular attention. The temperature of the gas where it issues from the soil is in some parts so high, that it is impossible to hold the hand for a minute at some distance over it: and there takes place at the same time a great condensation of vapour. In one spot the thermometer of Fahrenheit rose to 160°, but in others it did not rise higher than 70. Where the temperature of the gas was high, I observed that the edges of the fissures through which it issued were thickly studded with small groups of crystals of sulphur; where, however, the temperature was low, the quantity of sulphur was small, and the surfaces of the surrounding bodies were covered with the sulphates of lime, alumine, or iron, either crystallized or amorphous. This difference appeared at first somewhat extraordinary; but I was soon enabled to explain the cause from which it originated. Having carefully watched the escape of the gas, where of the temperature of 170°, I found that the following change took place.

A portion of the sulphuretted hydrogen where it came in contact with a mass of lava placed over the fissure, and of course freely exposed to the atmospheric air, became as it were condensed, forming, in a very short period of time, a transparent globule of fluid, possessing a highly refractive power. This continued to increase in size, until it seemed about to fall from the stone, when very minute particles, of a bright yellow colour, were seen to be revofving in the midst of it with considerable velocity. One of these particles now attached itself to the point of the stone from which the globule was suspended; to this others were seen rapidly to adhere, and in this manner a small prismatic crystal of sulphur was speedily produced. In the mean time the water evaporated, leaving the crystal in a state of great purity. I found that this process was much accelerated where the fissure took an oblique direction, evidently in consequence of the greater extent of surface exposed to the influence of the gas: and I also ascertained that the sulphur, thus produced, was either amorphous or crystallized, according to the rapidity with which the process was carried on; and, therefore, in accordance with one of the well-known laws of crystallization.

Where the gas issued at a comparatively low temperature, these phenomena were not observed: the change produced in surrounding bodies appeared to be but slowly and imperceptibly conducted; the quantity of sulphur was inconsiderable, while the sulphates, on the other hand, were abundant.

It will now be necessary to attempt to give the rationale of these processes.

In the first instance, we may presume, where the gas is combined with a great quantity of caloric, that, on coming in contact with the atmosphere, its vapour will not only be condensed, but its hydrogen will unite with a portion of oxygen to form an additional quantity of water; while its sulphur, being at the same time set free, will be deposited in a solid state upon the surfaces of surrounding bodies. If the temperature of the gas be low, the quantity of water which it holds in suspension will be inconsiderable; and probably its elastic tension may be precisely equal to that of the atmosphere. Under these circumstances, there will be no condensation: and at the same time, in consequence of the lowness of the temperature there will be no tendency to a union between the hydrogen of the gas and the oxygen of the atmosphere, although the latter will freely unite with the sulphur. Hence, then, we may expect that sulphuric acid, and not sulphnr, will be produced; and this, combining with salifiable bases, will give rise to the formation of those salts of which I have already spoken. The latter process appears to require some time for its completion; but so extensive is it that the whole soil of the Solfatara abounds in the sulphates, and the sulphate of alumine is readily obtained by simply washing the earth.

The crater of the Solfatara may be said to have an atmosphere of sulphuretted hydrogen; for the action of the gas is manifest on all surrounding bodies. Even the tufaceous lava is decomposed by it: the iron which this contains is converted into a sulphate, and thereby becomes soluble, so that, this having been removed by successive rains from the sides of the surrounding hills, it has ceased to give them their characteristic colour, and they now appear of a chalky whiteness, the earthy salts alone being left. It is on this account that these hills still retain the name of "*Colli Leucogei*," which appears to have been bestowed on them by the ancients.

It has been said by some travellers of reputation, that the soil of the crater of the Solfatara is even now but a hollow crust spread over an abyss; and the guides do not fail to turn this marvellous statement to profit. It is a fact that, when a heavy mass of lava is projected with some force upon the ground, a hollow rumbling sound is heard, as if the individual were standing over a vault; but the explanation of this phenomenon, with which I confess I was at the time very much struck, is far different from that which is commonly assigned. It is not heard to an equal degree in every part of the crater, but only in these spots where the disintegrating process is considerably advanced, a circumstance which might at once have sufficed to shew the futility of the vulgar opinion. Wherever there is but a slight adhesion between the earthy materials entering into the composition of a soil, that is to say, where these are loose, but at the same time so compact as not to yield easily to a violent shock, the reverberation, which is nothing more than the diffusion of the sound over a larger space, freely penetrated with air, will always be heard, and give rise to that peculiar impression which has been described. This fact I verified on the summit of the Puy de Dôme in Auvergne, during the autumn of 1828, where the hollow sound was still more remarkably distinct than in the crater of the Solfatara. I also frequently met with the same phenomenon in many parts of the Alps of Switzerland and Savoy.

---

*Oily Appearance of Dew.* Several gardeners in the environs of Rotterdam have observed, for some time, that the dew covering the leaves of plants in the morning, instead of being clear and thin as usual, has had an oily appearance, and sticks to the fingers. This change is said to have been simultaneous with the appearance of the cholera; and the question has been asked, whether it may not have been occasioned by the same atmospheric states which predispose to an attack of cholera.—*Gazette Med.*

